# Induction of B-cell lymphoma by UVB Radiation in p53 Haploinsufficient Mice

**DOI:** 10.1186/1471-2407-11-36

**Published:** 2011-01-26

**Authors:** Nahum Puebla-Osorio, Yasuko Miyahara, Sreevidya Coimbatore, Alberto Y Limón-Flores, Nasser Kazimi, Stephen E Ullrich, Chengming Zhu

**Affiliations:** 1 Department of Immunology and the Center for Cancer Immunology Research, The University of Texas M.D. Anderson Cancer Center, Houston, Texas, 77030, USA; 2 The Graduate School of Biomedical Sciences, Houston, Texas, 77030, USA; 3 Department of Hematology, Kyoto City Hospital, 1-2 higashitakada-cho, mibu, Nakagyo-ku, Kyoto, Japan 604-8845; 4 Escuela Nacional de Ciencias Biológicas-IPN, Prol Carpio y Plan de Ayala S/N, Mexico D.F., Mexico

## Abstract

**Background:**

The incidence of non-Hodgkin's lymphoma has increased over recent years. The exact etiology of lymphoma remains unknown. Ultraviolet light exposure has been associated with the development of internal lymphoid malignancies and some reports suggest that it may play a role in the development of lymphoma in humans. Here we describe the characterization and progression of lymphoma in p53 heterozygous mice exposed to UVB irradiation.

**Methods:**

UVB-irradiated p53^+/- ^mice developed enlargement of the spleen. Isolated spleen cells were transplanted into Rag deficient hosts. The UV-induced tumor cells were analyzed by flow cytometry. The tumor cells were tagged with GFP to study their metastatic potential. SKY and karyotypic analysis were carried out for the detection of chromosomal abnormalities. Functional assays included in vitro class switch recombination assay, immunoglobulin rearrangement assay, as well as cytokine profiling.

**Results:**

UVB-exposed mice showed enlargement of the spleen and lymph nodes. Cells transplanted into Rag deficient mice developed aggressive tumors that infiltrated the lymph nodes, the spleen and the bone marrow. The tumor cells did not grow in immune competent syngeneic C57Bl/6 mice yet showed a modest growth in UV-irradiated B6 mice. Phenotypic analysis of these tumor cells revealed these cells are positive for B cell markers CD19^+^, CD5^+^, B220^+^, IgM^+ ^and negative for T cell, NK or dendritic cell markers. The UV-induced tumor cells underwent robust in vitro immunoglobulin class switch recombination in response to lipopolysaccharide. Cytogenetic analysis revealed a t(14;19) translocation and trisomy of chromosome 6. These tumor cells secret IL-10, which can promote tumor growth and cause systemic immunosuppression.

**Conclusion:**

UV-irradiated p53^+/- ^mice developed lymphoid tumors that corresponded to a mature B cell lymphoma. Our results suggest that an indirect mechanism is involved in the development of internal tumors after chronic exposure to UV light. The induction of B cell lymphoma in UV-irradiated p53 heterozygous mice may provide a useful model for lymphoma development in humans.

## Background

The incidence of non-Hodgkin's lymphoma has increased over recent years, an increase that cannot be totally explained by improvements in diagnosis or reporting. The exact etiology of lymphoma remains unknown but viral infection, chronic antigen stimulation, and/or immunosuppression, either primary or acquired immunodeficiency, may all contribute to the occurrence of lymphoma [1]. Some reports suggest that exposure to the UV radiation in sunlight may play a role in the development of lymphoma in humans [2]. This conclusion is based primarily on epidemiological data showing a geographic correlation between sunlight exposure and lymphoma incidence (i.e., a latitude gradient) [3-7]. However, not all the reports supported a link between sunlight exposure and lymphoma development [8-11], some findings indicate an inverse association between the solar UVB exposure and the occurrence of non-Hodgkin lymphoma [12-15]. The rise in lymphoma incidence parallels the dramatic rise in melanoma incidence, and patients with non-Hodgkin's lymphoma or chronic lymphocytic leukemia are at a higher risk of developing skin cancer [16,17].

Studies with experimental animals suggest a strong correlation between UV exposure and lymphoma development [18,19]. Animal experiments offer the distinct advantage of controlled irradiation with defined UV light sources, without the complications that arise from exposure to any other environmental carcinogens or toxins, nor the complication of recall bias. We previously reported that UV irradiation augments lymphoid malignancies in mice with one functional copy of wild-type p53. In that study we demonstrated that UV irradiated p53 heterozygous mice developed lymphoid tumors at a much higher rate (88% of irradiated mice developed tumors) than found in un-irradiated animals (6% spontaneous tumor rate). Sequencing data indicated that the UV-irradiated p53^+/- ^mice retained the non-mutated p53 allele, suggesting loss of heterozygosity did not play a role in the induction of this tumor [19].

The biological effects of UV exposure are well known. UVB, wavelengths in the 280-320 nm range of the solar spectrum, can induce a wide variety of adverse effects. Chief among them are sunburn, inflammation, premature ageing of the skin, the induction of non-melanoma skin cancer and the induction of immune suppression (reviewed in [20]). UVA (320-400 nm) has been suggested to be important in melanoma induction [21]. It is clear that the adverse effects of UV exposure are not solely limited to the skin; how UV exposure influences lymphoma development in humans, however, is still far from being completely understood. Here we analyzed the UV-induced lymphoid malignancies that arise in mice with one functional copy of p53. We have previously analyzed UV-induced lymphoid malignancies by histopathological examination of formalin-fixed tissues. To be able to study the biology of the tumors we needed to generate tumor cell lines from UV-irradiated *p*53^+/- ^mice. We exposed *p*53^+/- ^mice to solar simulated UV radiation for a period of 30 weeks and then injected spleen cells into immune deficient Rag2^-/- ^mice. We generated a number of transplantable tumor cells lines, some of which were further adapted to grow in vitro. We found that UV-irradiation of *p*53^+/- ^mice resulted in the induction of B-cell tumors (CD5^+^, CD19^+^, B220^+^, IgM^+^) that grew progressively in immune deficient mice, but not in immune competent animals, and grew at low rate in UV-irradiated C57Bl/6 mice. We suggest the induction of B cell lymphomas by UV radiation in *p*53^+/- ^mice may serve as a useful animal model for lymphoma development.

## Methods

### Mice

Specific pathogen-free *p*53 knockout mice were backcrossed onto a C57Bl/6 background [19]. Specific pathogen-free Rag2^-/- ^mice were obtained from Dr. Fred Alt (Howard Hughes Medical Institute, Children's Hospital, Boston, MA). All mice were housed in a pathogen-free barrier facility. The Institutional Animal Care and Use Committee approved all procedures.

### UV irradiation

A 1000 W Xenon UV solar simulator equipped with a Schott WG-320 atmospheric attenuation filter and a visible/infrared band-pass filter (Oriel, Stratford, CT) was used to irradiate the mice. The intensity and spectral output was measured using an Optronics Model OL-754 scanning spectrophotometer (Optronics Lab, Orlando, FL) [22]. Seventeen p53 heterozygous mice were divided in two groups of 8 and 9 mice each for UV irradiation. The mice were shaved on the dorsal skin and were kept individually in a Plexiglas container; then, they were irradiated three times a week during 10-15 minutes for a period of 30 weeks. During this procedure the mice remained conscious all the time.

### Transplantation

Cells (2.5 × 10^6^) from enlarged spleens of the irradiated mice were injected subcutaneously into each flank of a Rag2^-/-^, wild type and/or UV irradiated (10 kJ/m^2^, 3 times per week for 12 weeks) C57Bl/6 mouse. Mice were monitored daily for tumor development. The tumors were excised and analyzed by flow cytometry and histopathology.

### Flow cytometric analysis

Cell surface molecule expression was analyzed by flow cytometry using a BD FACS-Calibur (BD Biosciences, San Jose, CA). Fluorescently-labeled monoclonal antibodies to mouse CD1d, CD3, CD4, CD5, CD8a, CD11b, CD16/32, CD19, CD23, CD25, CD38, CD40, CD43, CD44, CD45R/B220, CD49b, CD62L, CD80, CD86, CD90, CD117, CD123, CD127, CD138, IgM and IgD, GR-1 were purchased from commercial sources (Becton-Dickinson, Mountain View, California, and eBioscience, San Diego, California).

### In vitro class switch recombination assay

The cells were stimulated with lipopolysaccharide (LPS) (Sigma, St. Louis, MO), IL-4 (10ng/ml) plus CD40L (10 ng/mL), and a combination of CD40L/TGFβ (10 ng/mL each, all from PeproTech Rocky Hill, NJ). The cells were analyzed 96 hours later by flow cytometry using a BD FACS-Calibur or BD LSR II (Becton-Dickinson, Mountain View, California).

### Southern blotting analysis

High molecular weight DNA samples were prepared from the tumor, digested with EcoR1 and hybridized with a J_H _probe using Gene images AlkPhos Direct Labeling and detection System (Amersham Biosciences, Buckinghamshire, UK).

### Cytogenetic analysis

Tumor cells were cultured in the presence of Colcemid. Metaphases were analyzed with trypsin-Giemsa stain and with whole chromosome painting probes (Carlsbad, CA, USA). To analyze potential chromosomal translocations, the metaphases were also used for spectral karyotyping analysis (SKY; Applied Spectral Imaging, Inc. Vista, CA).

### Cell culture and lentiviral vector transduction

Tumor cells (1 × 10^5^) were incubated with lentiviral vectors. The number of green fluorescent protein (GFP) positive cells was analyzed by flow cytometry at multiple time points after transduction. Highly expressing GFP bearing cells were isolated by cell sorting (BD FACS-Aria, BD Biosciences, San Jose, CA).

### Cytokine determination

Cytokine secretion of the tumor cells into the supernatant fluid was measured by the multiplexed mouse cytokine Ten-Plex Antibody Bead system (BIOSOURCE, Camarillo, CA) and the Luminex 100 fluorescence detection system (Austin, TX). Cytokines analyzed by this system include: IL-1beta, IL-2, IL-4, IL-5, IL-6, IL-10, IL-12, GM-CSF, Interferon-gamma and TNF-alpha.

## Results

### UV exposure induced non-skin tumors in p53 heterozygous mice

To obtain a maximal probability of tumor development[19], we exposed 17 p53 heterozygous mice to 10 kJ/m^2 ^of UVB radiation, 3 times a week, during 30 weeks. At the end of this period, the animals were sacrificed. Analysis of necropsy showed severe skin irritation at the irradiated area and 15 out of 17 irradiated mice developed splenomegaly and half of which also developed enlarged mesenteric lymph nodes. To assess their tumorigenic potential, cell suspensions (approximately 2.6 × 10^6^) were prepared from the enlarged spleens and lymph nodes, and were injected subcutaneously into an immune deficient Rag2^-/- ^mouse. When tumors were evident they were excised and used for cell culture and histology (data not shown). A cell suspension sample from a tumor was re-injected into 3-4 Rag2^-/- ^mice. While the cells transplanted into the Rag2^-/- ^mice showed progressive growth, these cells were unable to grow ex vivo after the first in vivo passage. However, we were able to establish a cell line capable of growing in vitro after two to three additional passages through Rag2^-/- ^mice. At each successive passage through Rag2^-/- ^mice the latency period decreased, and after three passages subcutaneous tumors were noticeable at one-week post injection. This procedure was carried out several times and in all cases tumors were consistently generated in Rag2^-/- ^mice. However, only cells originated from the enlarged spleens were able to grow progressively in vivo and in vitro, whereas cells originated from the enlarged mesenteric lymph nodes were not able to survive in vitro. We were able to originate three cell lines that were able to grow in vivo and in vitro.

### UV-exposed mice developed a matured B-cell tumor

**Figure 1:**
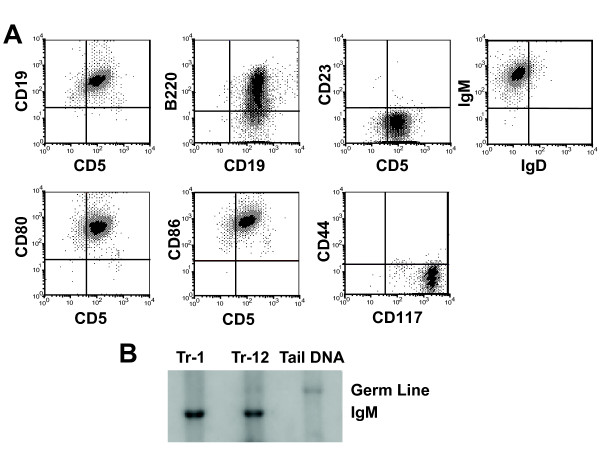
**Tumors that grow progressively in the Rag2**^**-/- **^**mice show a B cell phenotype**. A. Increased forward and side scatter was used to gate out the large tumor cells, and they were then stained with a variety of makers to determine their cell surface phenotype. B. Southern blotting indicates that the UV-induced B cells tumors have a rearranged immunoglobulin heavy gene. DNA was isolated from two variants of UVBL-1 (Tr-1 and Tr-12). Both variants expressed a re-arranged IgM heavy gene compared to genomic DNA isolated from non-lymphoid tissue (mouse tail skin).

**Figure 2:**
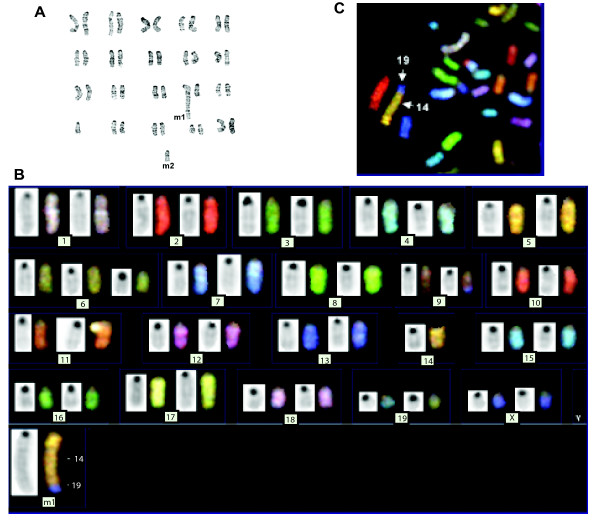
**Analysis of chromosomal abnormalities in the UV-induced B cell lymphoma**. A. G banding of metaphase spreads was prepared and indicated two abnormalities, an increase in size of chromosome 14, suggesting a translocation, and a small acrocentric chromosome (M2). B and C: Multi-color FISH confirms a translocation between chromosomes 14 and 19 and trisomal chromosome 6.

Phenotypic analysis was carried out using flow cytometry on the tumor cells that grew in Rag2^-/- ^mice. All the cells were included in this analysis, and we avoided selecting the cells by size. These tumor cells were positive to the surface markers CD19, B220, CD5 and IgM, and also expressed the co-stimulatory molecules CD80, CD86 as well as CD117 (Figure 1A). The cells were negative for the T cell specific markers CD3, CD4, CD8 and Th1.2; and were negative for the markers normally found on NK cells (NK1.1, CD49b) or dendritic cells/macrophages (CD11b). Moreover, the cells were also negative to surface markers expressed on immature B cells (CD43, BP-1, CD127). Southern blotting analysis showed a rearrangement of the immunoglobulin heavy chain in the DNA of the tumor cells as compared to the non-lymphoid DNA obtained from a Rag mouse, further supporting the conclusion that the tumor cells are indeed B-cells (Figure 1B). Using G-banding on metaphase spreads from the tumor cells we found a chromosomal translocation on chr14, and a small acrocentric chromosome. Spectral karyotyping (SKY) analysis confirmed the translocation was between the chromosomes 14 and 19 in all three cell lines studied (Figure 2 shows the representative results of one cell line). The translocated region was located proximal to the telomere on chromosome 19. SKY analysis also showed a trisomy on chromosome 19 (data not shown), with a likely complete translocation to chromosome 14. SKY analysis also showed a trisomy on chromosome 6 (Figure 2). The recurrent chromosomal translocation in all three cell lines strongly implies a role in tumorigenesis. However, the molecular details regarding the potential genes involved remain to be determined.

### The UV transformed B cells are stable and undergo robust class switch recombination (CSR)

**Figure 3:**
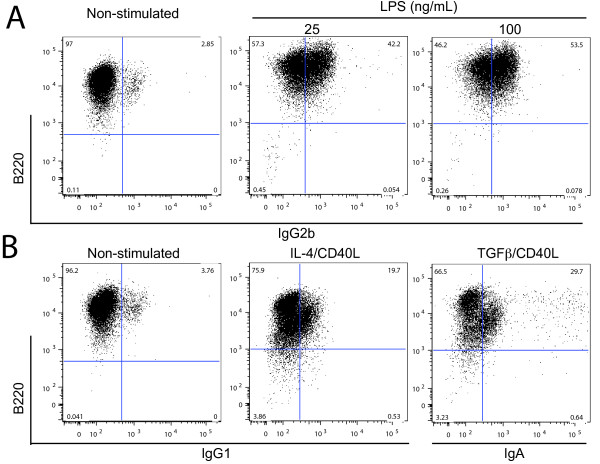
**UVBL-1 cells exhibit a robust CSR in response to LPS stimulation**. A. Flow cytometry analysis of untreated, 25 and 100 ng/ml LPS treated UVBL-1 cells undergo class switch recombination to IgG2b; B. Untreated, IL-4^+^CD40L and CD40L^+^TGFβ treated UVBL-1 cells class switch to IgG1 and IgA respectively.

To further explore whether the properties of these tumor B cells are typical of the B cell lineage, we examined their ability to undergo CSR in response to various stimuli [23]. The transformed B cells were kept in culture for 3-5 days and analyzed for spontaneous CSR. The cells with low spontaneous switching to IgG1, IgG3 and IgG2b (Figure 3) were sub-cloned by limiting dilution; the sub-cloned cells were stimulated with 25, 50 and 100 ng/mL of LPS. These experiments were performed at least six times. As shown in Figure 3A, LPS induced a robust CSR in these cells in a dose-dependent fashion from IgM to IgG2b at frequencies up to 53%. However, modest CSR levels were observed after these transformed B cells were stimulated with IL-4 and CD40L or CD40L and TGFβ, with frequencies of 19% to IgG1 and 29% to IgA, respectively (Figure 3B). The robustness of class switch recombination confirms the mature B cell phenotype of the transformed B cells, and the spontaneous CSR may be the result of a deregulated CSR enzymatic mechanism in this tumor cells [24].

### The UV-induced tumor B cells are immunogenic

One of the hallmarks of UV-induced skin tumors is the fact that they are highly antigenic since they are recognized and destroyed by a normal immune system, therefore these cells only grow progressively in immune-deficient mice [25]. In addition, UV-induced tumors will grow in mice exposed to a subcarcinogenic dose of UV radiation. This is due to the fact that UV exposure activates the induction of T regulatory cells that control the growth of UV-induced tumors in the UV-irradiated host [26,27]. To determine the growth patterns of the UV-induced B cell tumor and determine its antigenicity, the transformed B cells were injected into three groups of mice, normal immune-competent C57BL/6 mice, C57BL/6 mice exposed to a chronic course of UV radiation (10 kJ/m^2^, 3 times a week for 12 weeks), and immune-deficient Rag2^-/- ^mice (Table 1). Progressive tumor growth was only noted when the lymphoma cells were injected into the Rag2^-/- ^recipients (100% tumor incidence), significantly lower tumor incidence occurred when the cells were transplanted into wild-type mice (7%, p < 0.0001 vs. Rag2^-/- ^control, Fisher exact test). Lower but not significant tumor growth was noted in the wild type mice exposed to UV radiation after the injection of the lymphoma cells (13%, p < 0.0001 vs. Rag2^-/- ^control, Fisher exact test). These data indicate that like UV-induced skin tumors the UV-induced tumor B cells are antigenic, but unlike UV-induced skin tumors, these cells are not recognized by UV-induced T regulatory cells.

**Table 1:** Growth of UV-induced B cell tumors in immune-deficient but not wild type mice

Recipient Mice*	Number of mice	Number with tumor	p‡
Rag2^-/-^	15	15	_
C57Bl/6	15	1	0.0001
UV-irradiated C57Bl/6†	15	2	0.0001

* 2 × 10^6 ^tumor cells were injected into the subcutaneous space of the recipient mice. Tumor growth was monitored daily. Mice were sacrificed when the tumor volume exceeded 1 cm^2^.

† 10 kJ/m^2 ^UVB radiation, 3x per week for 12 weeks prior to tumor transplantation

‡ p values determined by Fisher's exact test comparing tumor growth in immune deficient Rag2^-/- ^vs. growth in wild type or UV-irradiated wild type mice.

### The UVB induced p53 heterozygous B lymphoma cells are metastatic

**Figure 4:**
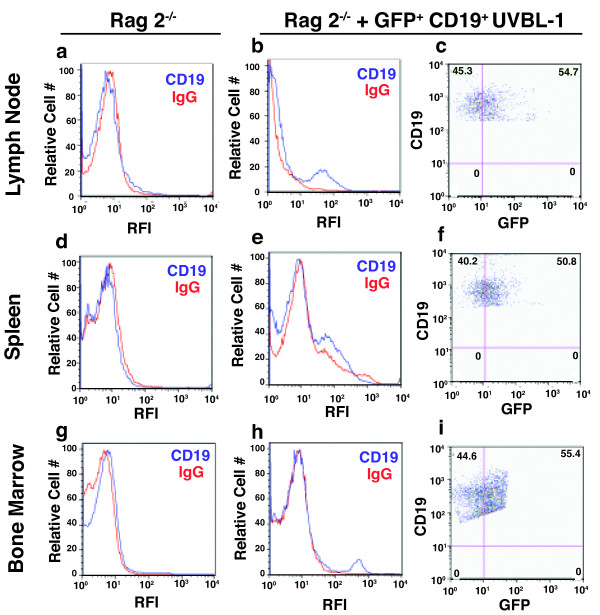
**Metastatic behavior of the CD19**^**+ **^**B cell lymphoma**. CD19^+ ^UVBL-1 cells were transfected with GFP and injected subcutaneously into the flanks of Rag2^-/- ^mice. Metastasis was analyzed 11, 18 and 27 days post injection. A: Background staining in the lymph nodes of Rag2^-/- ^mice; isotype staining in red; CD19 staining in blue. B: CD19 staining in the lymph nodes of Rag2^-/- ^mice injected with UVBL-1; isotype staining in red; CD19 staining in blue. C: Double staining showing expression of CD19 by GFP positive cells Lymph node metastasis was analyzed 11 days post implantation. D: Background staining in the spleens of Rag2^-/- ^mice. E: CD19 staining in the spleens of Rag2^-/- ^mice injected with UVBL-1. F: Double staining showing expression of CD19 by GFP positive cells. Bone marrow metastasis was analyzed 27 days post injection. G: Background staining in bone marrow of Rag2^-/- ^mice. H: CD19 staining in the spleen of mice injected with UVBL-1. I. Double staining showing expression of CD19 by GFP positive cells. Bone marrow metastasis was analyzed 27 days post injection. All other tissues examined (thymus, lung, live kidney) were negative.

UV-transformed B cells that resulted from exposing p53 heterozygous mice to UVB light were transfected with a vector expressing green fluorescent protein (GFP). The cells were sorted for both CD19 and GFP. Approximately 1 × 10^7 ^CD19/GFP double positive tumors cells were injected subcutaneously into 10 Rag2^-/- ^mice. Tumor development was assessed in these animals and the tumor bearing Rag2^-/-^mice were sacrificed at various time points after the injection. Flow cytometry was utilized to determine the numbers of GFP/CD19 double positive cells in various lymphoid tissues (Figure 4). Cells from the spleen and bone marrow of normal Rag2^-/- ^mice were used as control. CD19^+ ^cells were absent in all lymphoid tissues of the non-injected Rag2^-/- ^mice (Figure 4A, D, G). The CD19^+^, GFP^+ ^cells were only found in the subcutaneous tumor 4 days post injection. On day 11 these cells were found in the draining lymph nodes (Figure 4B &4C), but were absent in the spleen or bone marrow. Two populations of CD19^+ ^cells were found in the lymph nodes (Figure 4C), one was negative for GFP (45.3%), and the second was positive for GFP (54.7%). These results suggested that although our vector was "leaky", and since Rag2^-/- ^mice do not have any CD19 cells in their lymph nodes, the UV-transformed B cells metastasized to the lymph nodes after 11 days post implantation. The metastatic cells not only persisted in the lymph nodes at day 18 (data not shown), but were also found metastasized to the spleen (Figure 4 E &4F). The metastatic cells were still detected in the spleen and lymph nodes at 27 days post implantation, and during this period these cells were also detected in the bone marrow (Figure 4H &4I). To determine whether the metastatic cells had migrated to other organs, we analyzed cell suspensions from the thymus, lung, liver and kidney, and found no evidence of these cells in any of these organs. These results indicate that the UV-induced B cell tumor cells were capable to metastasize to the bone marrow, spleen and lymph nodes, but not to any other tissue.

### The UV-induced tumor B cells are rich in IL-10 secretion

**Figure 5:**
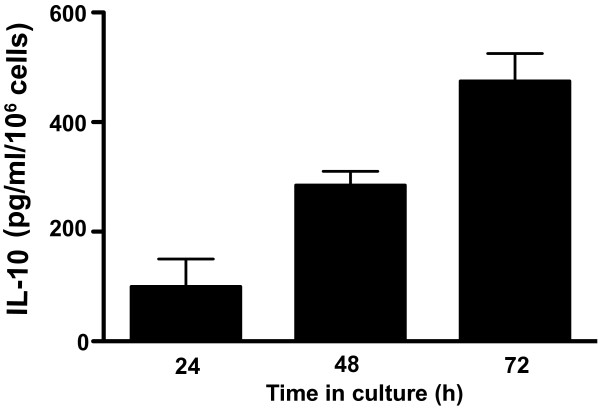
**IL-10 secretion by the UV-induced B cell lymphomas**. The cells were grown in vitro without any further stimulation. Supernatants from duplicates of three Petri dishes were collected at 24, 48 and 72 hours and were analyzed individually. Each column represents the average in IL-10 concentration of 6 samples. The only cytokine detected in the supernatant fluid was IL-10. The bar indicates the standard error of the mean (SEM).

To establish cytokine profiles, supernatant from cultured UV-induced tumor B cells were collected at various time points after the initiation of culture (24 to 72 hours). The production of a variety of cytokines by the cultured cells was analyzed using the Luminex system. Our results showed that the un-stimulated tumor cells only secreted IL-10 when cultured for 24 and 72 h in complete RPMI medium (Figure 5). These results indicate that IL-10 producing cells may have an increased advantage to promote their metastasis to other organs as observed in our analyses.

## Discussion

We characterized the lymphoid tumors that arise in UV-irradiated p53^+/- ^mice. No evidence of T cell tumors was found in this study. This may reflect the different way in which this study and our previous work was carried out. Our observation that UV exposure induced T cell tumors was based on a retrospective study in which histopathological analyses of paraffin-embedded tissue was used to determine the phenotype of the tumor [19]. Here, we generated tumors by injecting spleen cells isolated from UV-irradiated p53 heterozygous mice into Rag2-/- mice. The tumor cells were subjected to several passages in vivo until we were able to generate stable tumor cell lines that could grow in Rag2^-/- ^mice and in culture. From these experiments we obtained three cell lines that grew in vivo but only one was also able to grow in vitro. It is possible that our "culture" favored the growth of B cells but not T cells. In one series of experiments, we phenotyped each successive in vivo passage, and in one of the earlier passages, we noted the presence of some T cell receptor positive cells (which by definition must be of tumor origin since the Rag2^-/- ^mice cannot rearrange the T cell receptor). These cells were purified by cell sorting and then injected into Rag2^-/- ^mice. No T cell tumors developed. This suggests that abnormal cells may have been present, but were not capable of growing progressively upon further passage in vivo.

It is interesting to compare our results to those previously published by MacPherson and colleagues [28]. In their study, transgenic mice were constructed with a point mutation in the murine p53 gene (serine 23 was replaced with alanine). These mice, which displayed defective apoptosis, had a reduced life span and most developed tumors. However, unlike the tumor spectrum found in p53^-/- ^mice (predominately thymic lymphomas and sarcomas), the most common tumors found in homozygous p53 Ser23 mice were of B cell origin. The authors suggest that the long latency period to tumorigenesis in the p53 Ser23 mice provides the time required to acquire additional genetic changes to develop B cell tumors. We also note a long latency period for tumor development in our system, and as mentioned below, we suggest UV-induced inflammation may play a significant role.

Chromosomal analysis revealed a t(14:19) translocation, which may play a role in UV-induced lymphomagenesis. However, to the best of our knowledge, a similar translocation has not been reported in lymphoma in the mouse. We analyzed the translocated region of chromosome 19 and found that this region contains the apoptotic genes Caspase 7 and programmed cell death 4, and the DNA cross-link repair 1a gene (SNM1a) which is involved in G1 cell cycle checkpoint downstream of ATM[29]. We suspect the t(14;19) translocation contributes to the deregulation of these and other genes and probably plays an important role in the oncogenic process in these cells. In addition, trisomy of chromosome 6 has been reported in chemically induced malignant skin tumors in TG.AC mice, which carry the v-Ha-ras transgene[30], and a similar imbalance has been reported in both benign and malignant skin tumors in the mouse [31]. Therefore, it is likely that the trisomy of chromosome 6 observed in our model is the result of the chronic exposure to UVB and probably it is also a contributing factor to the transformation of the UV-induced tumor B cells. We are currently exploring the possible deregulated oncogenes/tumor suppressors in these tumor cell lines.

What are the signals involved in the maintenance and proliferation of these tumors? Simple loss of heterozygosity does not appear to be an adequate explanation because when the B cell lymphomas were sequenced, we found they retained a normal wild-type p53 allele (data not shown). We favor the view that the immune suppression and chronic inflammation induced by UV exposure are involved. As noted above, the B cell lymphomas are highly antigenic. From our previous study we know that p53^+/- ^mice are immune competent and UV exposure will induce immune suppression in these mice [19], so it is reasonable to assume that the immune suppression induced by chronic UV exposure provides a window that will allow these antigenic cells to grow in the UV-irradiated p53^+/- ^mice. While it has been argued that an inverse association of sun exposure with NHL risk is related to the protective effects of vitamin D synthesis by UV radiation [15,32-36]; we know that the induction of systemic immune suppression following UV exposure of the skin is associated with the release of immune modulatory factors and immune suppressive cytokines. These may have direct relevance to the results reported here. The first is IL-10. UV-irradiation of the skin induces keratinocytes to secrete IL-10 [37,38] and IL-10 can be found in the serum of UV-irradiated mice [39]. Moreover, IL-10 promotes the growth and differentiation of human B cells [40] and has been shown to promote the growth of B cell lymphomas [41,42]. Serum IL-10 is also considered to be a negative prognostic indicator for the survival of patients with non-Hodgkin's lymphoma [43]. The second is platelet-activating factor (PAF). PAF is a phospholipid that plays a primary role in the induction of inflammation. As the name implies PAF activates a wide variety of cells, including platelets, monocytes, mast cells and polymorphonuclear leukocytes [44]. PAF is secreted by epidermal cells almost immediately following UV exposure [45] and serves as a transcriptional activator for a variety of genes including cyclooxygenase (COX)-2, IL-6 and IL-10 [46,47]. PAF also plays an essential role in UV-induced immune suppression. Blocking the binding of PAF to its receptor, with a series of selective PAF receptor antagonists, blocks UV-induced transcription of COX-2 and IL-10 and blocks UV-induced immune suppression [47]. Normal and leukemic B cells express the PAF receptor [48,49] and PAF has been shown rescue B cells from apoptosis [50].

Our results suggest the following scenario: the loss of a single p53 allele predisposes the B cells for transformation. Long-term chronic UV exposure then induces the release of inflammatory mediators by irradiated keratinocytes that promote tumor development. For example, UV-irradiated keratinocytes secrete macrophage migration inhibitory factor (MIF) [51], which can inhibit p53 tumor suppressor activity [52], thus resulting in a functional "loss of heterozygosity" in the UV-irradiated p53^+/- ^mice. Other UV-induced mediators of inflammation, such as PAF, may also play important roles in the process. First, PAF induces chronic inflammation, which appears to be associated with the induction of non-Hodgkin's lymphoma [53]. Second, PAF binds to receptors on B cells and protects them from apoptosis. PAF up-regulates the transcription of COX-2 and IL-10. This is important because the expression of either prostaglandin E_2 _or IL-10 has been associated with a poor prognosis in lymphoma patients [43,54]. In addition, both are immune suppressive, and we suggest UV-induced immune suppression (mediated by PAF, prostaglandin and IL-10), protects the developing antigenic B cell lymphoma from immune destruction. Our data provide a link between chronic UV exposure and the incidence of internal tumors in the 53 heterozygous background. Although there is no development of skin tumors in p53 heterozygous mice, somehow chronic UV exposure develops cellular transformation away form the site of exposure by a mechanism still under investigation.

## Conclusion

We characterized the lymphoid tumors that arise in UV-irradiated p53^+/- ^mice. The tumors were CD5^+^, CD19^+ ^B cell lymphomas that showed robust CSR capacity upon stimulation. The lymphomas secreted Il-10, which might help in the promotion of metastasis and lymphoma growth. Chromosomal analysis revealed a t(14;19) translocation and trisomy of chromosome 6. Sequencing analysis indicated that the remaining p53 allele was intact, suggesting that an indirect mechanisms is involved in the generation of these tumors. In the future, we intend to try several therapies to block the induction of the UV-induced B cell lymphomas in p53^+/- ^mice including PAF receptor antagonist PCA-4248 treatment [55] or anti-IL-10 antibody therapy [56]. If successful, it may provide support for the hypothesis that the association between sunlight exposure and the development of non-Hodgkin's lymphoma in humans is associated with chronic inflammation and the modulation of immune function by chronic UV exposure.

## Competing interests

The authors declare that they have no competing interests.

## Authors' contributions

NP: designed and performed experiments, interpreted results, drafted and revised the manuscript; YM, CS, AYL performed experiments, interpreted results, drafted the manuscript; NK performed experiments using animals; SEU and CZ designed and supervised the research, analyzed the data and wrote and modified manuscript.

All authors read and approved the final manuscript.

## Pre-publication history

The pre-publication history for this paper can be accessed here:

http://www.biomedcentral.com/1471-2407/11/36/prepub
